# A large-scale graph-augmented traffic dataset for data-driven spatio-temporal traffic analysis

**DOI:** 10.1038/s41597-026-07672-8

**Published:** 2026-07-14

**Authors:** David Maria-Arribas, Juan J. Pantrigo, Alfredo Cuesta-Infante

**Affiliations:** Department of Computer Science and Statistics, Universidad Rey Juan Carlos Tulipán s/n, 28933 Móstoles, Madrid Spain

## Abstract

This paper introduces a large-scale, high-resolution traffic dataset collected from over 5,000 sensors across the city of Madrid, Spain, spanning a ten-year period, from 2015 to 2024. Comprising more than 1.5 billion records, the dataset includes key traffic metrics such as intensity, occupancy, and average speed, aggregated at 15-minute intervals. It also features two distinct graph-based spatial representations of the sensor network, enabling advanced modelling of urban mobility. The dataset is designed to support a wide range of machine learning tasks, including spatio-temporal forecasting, representation learning, and transfer learning, while also serving broader applications in urban planning and smart city development. Its geographic diversity offers a valuable alternative to existing datasets predominantly sourced from California, enhancing model generalization and reducing regional bias. Additionally, traffic data from this dataset can be used as a covariate in related domains such as air quality control, supporting multi-modal approaches to urban sustainability.

## Background & Summary

Traffic management is a critical issue that significantly impacts citizens’ daily lives. According to TomTom traffic index^1^ in 2024, cities like Barranquilla (Colombia), Bengaluru (India) and London (United Kingdom) reported average travel times of 36 minutes for 10 km; i.e., an average speed of 16.6 km/h. Similarly, the INRIX Global Traffic Scorecard^2^ identified London as the most congested driving area in the UK, with drivers losing over 100 hours per year due to traffic delays, resulting in an estimated economic loss exceeding EUR 1,088 billion to the city. Beyond delays and economic costs, traffic congestion also affects housing prices^3,4^, environmental pollution, and consequently, public health and quality of life. The design and deployment of Traffic Management Systems (TMS) for urban mobility depend on a robust data-centric foundation^5^. However, the effective collection and utilization of traffic data are hindered by the heterogeneity of data sources (e.g., sensors, GPS, social media), real-time processing demands and sensor reliability. Additionally, resistance to change within traffic control centers can hamper the adoption of new, data-driven workflows, limiting the system’s potential impact. Moreover, quantifying return on investment in TMS is difficult, especially when compared to traditional infrastructure projects, making it harder to secure consistent funding from public agencies.

These challenges have recently been addressed with neural network models^6,7^, which are currently outperforming other methods in prediction, regression, and classification tasks with temporal and spatio-temporal data. A cornerstone of these approaches is a large, diverse and curated data set for training and testing the model.

Existing traffic datasets can be broadly classified in two types: long-distance traffic data (regional scale) and local traffic data (city scale). Long-distance traffic datasets capture flows between cities or across broader territories, supporting long-distance travel analysis, infrastructure planning, and regional connectivity studies. A widely adopted source of this kind is the Caltrans Performance Measurement System (PeMS) (https://pems.dot.ca.gov/) provides more than ten years of traffic records gathered from major interstate routes across California, USA. In contrast, local traffic datasets focus on traffic dynamics within cities such as intersections, traffic signals, and public transit corridors; and are typically used for real-time traffic control and urban mobility optimization. The UTD19^8^ dataset stands out for its comprehensive coverage over 40 cities worldwide, spanning nearly four years of urban traffic data.

In this paper we introduce GO-MO, a Graph augmented Open urban MObility dataset, extracted from the publicly available Open Data Portal of the City Council of Madrid (Spain). GO-MO comprises more than 1.5 billion records of three traffic-related metrics together with spatio-temporal data and metadata, spanning a ten-year period (2015–2024). Specifically, data is collected and aggregated at 15-minute intervals by a network of 5,157 sensors (induction loops and cameras equipped with computer vision systems).

GO-MO is derived from the operational traffic data published by the Madrid City Council. The official source documentation states that the traffic sensing infrastructure follows the AEN/CTN 199 framework, including data-quality procedures, and that the published measurements contain a validity/error control field^9^. This framework belongs to the Spanish national standardization system and is connected to a broader European and international standardization ecosystem through UNE and the international relations of CTN 199^10,11^. On top of these source-level controls, GO-MO adds a reproducible curation layer based on rule-based cleaning, out-of-range checks, missing-data characterization, and interpolation flags.

A new file is released on a monthly basis, containing approximately 11 million records. Thus, to the best of our knowledge, GO-MO becomes the largest local traffic dataset related to a single highly-populated city. Besides, we provide two graphs representing the routes between sensors and the road network respectively, constructed using data from OpenStreetMap and other providers of public geospatial information that enrich the dataset.

**Contributions**. (1) We introduce a large-scale open-source local traffic dataset comprising more than 1.5 billion records over ten years, which has been curated to remove missing values and errors, (2) we enrich the data with two different graph representations, (3) we also carry out an analysis of the spatio-temporal dynamics, (4) we provide a traffic forecasting baseline. (5) we discuss the advantages of such a large dataset from a single 3.5 million people city compared with a smaller dataset from many cities worldwide.

### Related work and datasets

In 2001, Chen *et al*.^12^ described the architecture and development of the Performance Measurement System (PeMS) for the California Department of Transportation (Caltrans). Such a system ingests 2 GB of data per day, holding at that moment 400 GB of data online. Thus, PeMS has been the source for several datasets used in recent traffic prediction studies. Yu *et al*.^13^ proposed modeling traffic data as a graph to capture both temporal and spatial dependencies using Graph Convolutional Networks (GCNs). In their study, they conducted experiments on two datasets. The first one, “PeMSD7”, was collected from California’s District 7 and is publicly available in two variants: PeMSD7 (Medium) and PeMSD7 (Large). For the latter, the authors constructed a weighted adjacency matrix representing the road network graph, where the weights are determined based on the pairwise distances between traffic monitoring stations. The second one, “BJER4” dataset, was collected from selected segments of Route 4 in Beijing but it is not publicly available due to confidentiality restrictions.

Li *et al*.^14^ proposed modeling traffic flow as a diffusion process on a graph and introduced the Diffusion Convolutional Recurrent Neural Network (DCRNN) framework. They constructed two datasets: “METR-LA”, which contains four months of traffic data collected from 207 sensors on highways in the Los Angeles area, and “PEMS-BAY”, which includes data from 325 sensors in California’s Bay Area; both sourced from PeMS.

Song *et al*.^15^ proposed the Spatial-Temporal Synchronous Graph Convolutional Network (STSGCN), which incorporates a novel spatio-temporal graph convolutional module designed to capture spatial and temporal dependencies at the same time. To evaluate their approach, the authors constructed four datasets corresponding to different districts from PeMS, namely “PEMS03”, “PEMS04”, “PEMS07”, and “PEMS08”. The traffic data is aggregated at 5-minute intervals. Unlike previous methods that rely on distance-based adjacency matrices, the authors constructed the graph structure based on the actual road network topology: two sensors are connected in the adjacency matrix if they are located on the same road segment. This approach results in a sparse graph, with the average node degree ranging from 1.0 in the PEMS07 dataset to 1.6 in the PEMS08 dataset.

Liu *et al*.^16^ focused on advancing the availability of high-quality data for large-scale traffic forecasting. They introduced “LargeST”, a benchmark dataset designed to facilitate the development and evaluation of models in this domain. LargeST significantly surpasses its predecessors in scale and coverage. The main dataset, referred to as “CA” (after California), includes traffic data collected from 8,600 sensors over a five-year period, spanning from January 2017 to December 2021. In addition, the authors released three regional subsets: “GLA”, comprising 3,834 sensors in the Greater Los Angeles area; “GBA”, with 2,352 sensors in the Greater Bay Area; and “SD”, the smallest subset, containing 716 sensors located exclusively in the San Diego region. To construct the adjacency matrix, the authors employed the Open Source Routing Machine (OSRM) (https://project-osrm.org/) to compute the shortest driving distances between sensors. To mitigate the computational cost of calculating all pairwise routes, they first computed pairwise Euclidean distances and then, for each sensor, calculated routing distances only to those within a 4 kilometer radius.

With respect to urban mobility, the UTD19 multi-city traffic dataset provides traffic data from 40 cities worldwide spanning almost 4 years, which adds up to 170 million data rows. Loder *et al*.^8^ pair this data with OpenStreetMap to explain how network topology drives the critical number of vehicles and the network’s traffic capacity.

The key features of these datasets are compared with ours in Table 1. GO-MO, a single city local traffic dataset, exceeds or matches long-distance datasets in terms of number of traffic sensors, day count, and data points, and it also compares favourably for UTD19, a city-scale dataset that collects information from 40 cities. Same can be remarked regarding the graphs provided with GO-MO. Our two graphs are similar or larger in number of nodes, edges and average degree to those reviewed above.

**Table 1:** Comparison of GO-MO with other large-scale traffic datasets.

Ref.	Dataset	Nodes	Edges	Deg.	TS	M	ST	ET	Res.	Data p.
Yu *et al*.^13^	PeMSD7(M)	228	1,664	7.3	228	6	01/05/2012	30/06/2012	0:05:00	2,890,000
	PeMSD7(L)	1,026	14,534	14.2	1,026	0	01/05/2012	30/06/2012	0:05:00	13,000,000
Li *et al*.^14^	METR-LA	207	1,515	7.3	207	3	01/03/2012	27/06/2012	0:05:00	7,090,000
	PEMS-BAY	325	2,369	7.3	325	3	01/01/2017	30/06/2017	0:05:00	16,940,000
Song *et al*.^15^	PEMS03	358	546	1.5	358	1	01/09/2018	30/11/2018	0:05:00	9,380,000
	PEMS04	307	338	1.1	307	0	01/01/2018	28/02/2018	0:05:00	5,220,000
	PEMS07	883	865	1.0	883	0	01/05/2017	06/08/2017	0:05:00	24,920,000
	PEMS08	170	276	1.6	170	0	01/07/2016	31/08/2016	0:05:00	3,040,000
**Liu** ***et al****.*^16^	CA	8,600	201,363	23.4	8,600	9	01/01/2017	31/12/2021	0:05:00	4,520,000,000
	GLA	3,834	98,703	25.7	3,834	9	01/01/2017	31/12/2021	0:05:00	2,020,000,000
	GBA	2,352	61,246	26.0	2,352	9	01/01/2017	31/12/2021	0:05:00	1,240,000,000
	SD	716	17,319	24.2	716	9	01/01/2017	31/12/2021	0:05:00	380,000,000
Loder *et al*.^8^	UTD19	n/a	n/a	n/a	23,541	n/a	n/a	n/a	0:05:00	170,000,000
**GO-MO (ours)**	with RG	5,157	135,428	52.52	5,157	8	01/01/2015	31/12/2024	0:15:00	1,523,167,456
	with RNG	35,304	69,362	3.92	5,157	8	01/01/2015	31/12/2024	0:15:00	1,523,167,456

For GO-MO, RG denotes the Routes Graph and RNG denotes the Road Network Graph, both formally described in the Graph generation section.

**Column Descriptions: Ref**.: Released (Publication Source), **Dataset**: Dataset name, **Nodes**: Number of nodes in the graph, **Edges**: Number of edges in the graph, **Deg**.: Average degree, **TS**: Traffic Sensors, **M**: Meta Information Count, **ST**: Start Time, **ET**: End Time, **Res**.: Data Resolution, **Data p**.: Data Points. n/a not available.

## Methods

The chosen approach for generating the GO-MO begins with the Extract-Load-Transform (ELT) process, which is particularly well-suited for handling large-scale datasets^17^. In this workflow, data is first extracted from the sources, then loaded into a relational database, and finally transformed through cleaning and feature engineering processes. After this step, two graphs are constructed representing routes between sensors and the road map respectively. Finally, we query the database to generate the CSV files that are ultimately published together with the graphs.

### Extracting and loading data

The data used to build this dataset were obtained from the Open Data Portal of the Madrid City Council^18^.

**Fig. 1:**
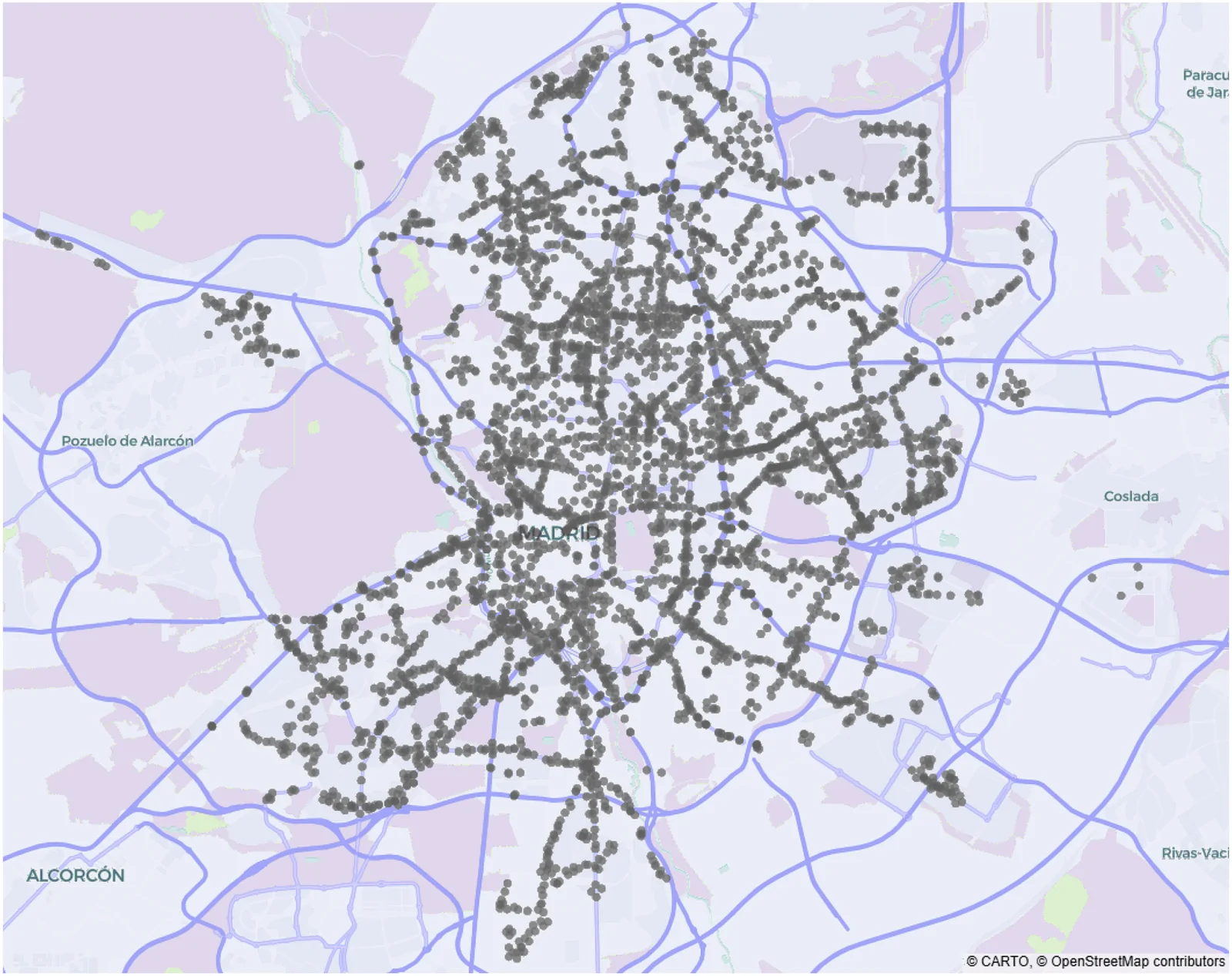
Madrid City Council traffic sensors locations.

The Madrid City Council manages 5,258 sensors, of which 5,157 actively reported data for this dataset. Figure 1 shows the locations of these 5,157 active sensors as black dots, and also shows the highways that distribute the most traffic (light blue lines) and the city’s green areas (as light red zones). The data source is licensed by the Madrid City Council and it permits both commercial and non-commercial use, provided that appropriate attribution is given to the City Council of Madrid^19^.

The Open Data Portal of the Madrid City Council publishes two CSV files each month: one with traffic data and another with sensor information. Readings of traffic_intensity, sensor_occupancy and avg_speed (see Table 2) are aggregated at 15-minute intervals and compiled into a CSV file which is published monthly; each file containing approximately 12 million records. These files have been published since January 20, 2013 at 20:15:00 (Madrid local time), but for the purpose of this paper we begin the compilation on January 1, 2015. The fields in these CSV files are described in Table 2. In addition, the data source also publishes a companion CSV file on a monthly basis that gives information about the sensors, whose fields are described in Table 3.

**Table 2:** Description of every field from the CSV data files provided by the source.

sensor_id	Integer sensor identifier.
entry_date	Timestamp of the measurement (Madrid time zone).
× element_type	Text field indicating sensor location: ‘URB’ for sensors on city streets,
	plus other 4 types for non-urban sensors (distribution rings, etc).
traffic_intensity	Number of vehicles in the 15-minute period, expressed in vehicles/h.
sensor_occupancy	Percentage of time the sensor was occupied by a vehicle.
avg_speed	Average speed (only available for C30 sensors).
× road_load	Road load, calculated from intensity and occupancy.
read_error	Quality flag indicating data reliability:
	N - no errors,
	E - some values are suboptimal,
	S - some values are completely erroneous and not aggregated.
× integration_period	Number of samples collected and aggregated in the interval.

× marks those that are not used in the CSV files published in GO-MO.

**Table 3:** Description of every field in the CSV sensor information files provided by the source.

id	Integer sensor identifier, that matches the sensor_id field in Table 2.
element_type	as in Table 2.
district	Administrative district of Madrid where the sensor is located.
internal_code	Internal code assigned to the sensor
name	Name of the sensor, including the name of the road where it is installed
utm_x	UTM 30 N × coordinate of the sensor location
utm_y	UTM 30 N y coordinate of the sensor location
longitude	WGS84 longitude of the sensor location
latitude	WGS84 latitude of the sensor location

**Fig. 2:**
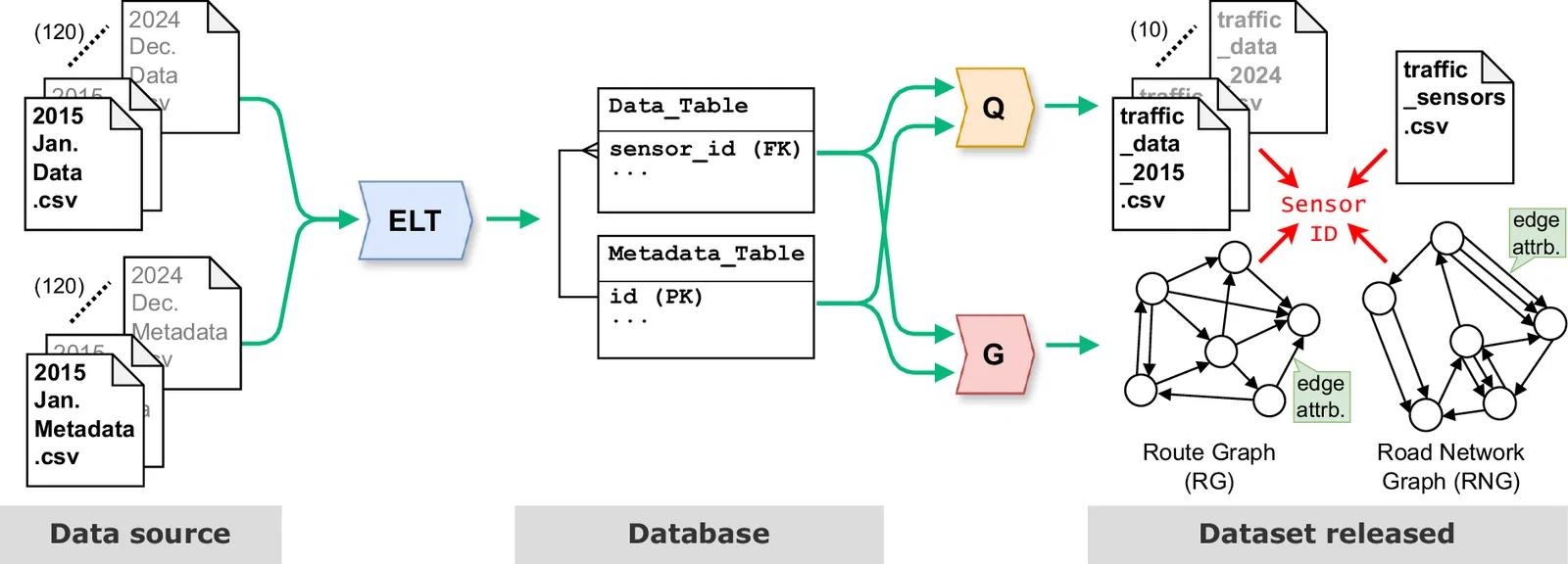
Process followed to transform the data sources into GO-MO. The ELT block represents the Extraction-Load-Transformation that produces a relational database out of the CSV files from the source. The Q block comprises the queries executed to obtain the CSV files published. The G block entails the production of the companion graphs RG and RNG.

To construct GO-MO we import all available traffic data and sensor information CSV files from January 1, 2015 up to December 31, 2024 into a relational database that has two tables, namely: traffic_data and traffic_sensors. Besides, the sensor_id field is a foreign key (FK) of traffic_data that corresponds to the id field, the primary key (PK) of traffic_sensors in a one-to-many relationship as shown in the central part of Fig. 2. The rest of fields in traffic_data come directly from Table 2; and similarly the fields in traffic_sensors are those in Table 3. Additionally, to enrich the available information about the sensors, we do reverse geocoding on each sensor’s location using the OpenStreetMap API. Thus, we identify the road segment nearest to each sensor, thereby determining the road on which the sensor is installed. Then, for such a segment, we extract the additional information: number of lanes, maximum speed limit, and the OpenStreetMap road name. These are stored as three additional fields in traffic_sensors, namely: lanes, max_speed, and osm_name. This metadata is further used to estimate the maximum road capacity, which supports the identification of out-of-range traffic intensity values.

The total number of rows in traffic_data is 1,523,167,456, with an average of approximately 12.28 million rows per month; where each row represents traffic data from a single sensor at a specific timestamp. Hence, to ensure efficient data management and querying, the main data table is partitioned by year and month, resulting in 120 partitions.

### Data cleaning

This stage involves two actions: (1) correcting errors and out-of-range data and (2) imputing missing values. The former happens in two cases: when the field read_error indicates it and when the values exceed the maximum possible value. The latter is detected in three possible ways depending on what happens to the row corresponding to that timestamp: it does not exist, it does not have a value, or it has a negative value, which encodes ‘absence of data’.

#### Errors and Out-of-range values

The city council provides the field read_error with three possible exclusive values:

- N: No errors in the sensor readings.
- E: Some of the collected values are suboptimal.
- S: Some values are erroneous and have not been integrated into the aggregated results.

Only 2,032,431 records out of the entire dataset ($1.334\times {10}^{-1}{\rm{ \% }}$) are marked with an error flag (S), while just 19 records ($1.25\times {10}^{-6}{\rm{ \% }}$) are labeled as suboptimal (E). Given their negligible number, we handle them as missing values and apply interpolation as explained below.

For detecting out-of-range errors in the traffic_intensity, we estimate the theoretical maximum traffic flow capacity of the road segment where each sensor is located according to the model introduced in^20^. With it, the maximum traffic intensity is computed using Eq. (1):

1 $${\rm{T}}{I}_{{\rm{m}}ax}=\frac{3600}{\frac{L+{d}_{{\rm{g}}ap}}{{v}_{{\rm{m}}ax}}+\tau },$$

where L=4.5 m is the average vehicle length, ${d}_{{\rm{g}}ap}=2.5$ m is the minimum distance between two consecutive vehicles, τ=1 is the minimum time headway between vehicles passing the same point, and ${v}_{{\rm{m}}ax}$ is the speed limit (m/s) for the road segment. These constants are based on values from the SUMO project^21,22^. We remark that the speed limit ${v}_{{\rm{m}}ax}$ is not provided by the data source. We use the geographic location of each sensor to identify its associated road segment and obtain the speed limit via the OpenStreetMap API. Hence, any traffic_intensity value exceeding the computed ${\rm{T}}{I}_{{\rm{m}}ax}$ is considered out-of-range error and it is corrected by setting it to the ${\rm{T}}{I}_{{\rm{m}}ax}$ value. Finally, avg_speed and sensor_occupancy are clipped to 100 because they represent percentage values.

#### Missing data

Regarding missing data, we identify two types:

1. *Completely missing rows* are those in which all three traffic metrics (intensity, occupancy, and average speed) are absent. They account for $8.5615{\rm{ \% }}$ of the entire database.
2. *Partially missing rows* are those where either one or two out of the three metrics are missing. These only represent the $5.680\times {10}^{-1}{\rm{ \% }}$ of the entire database.

**Fig. 3:**
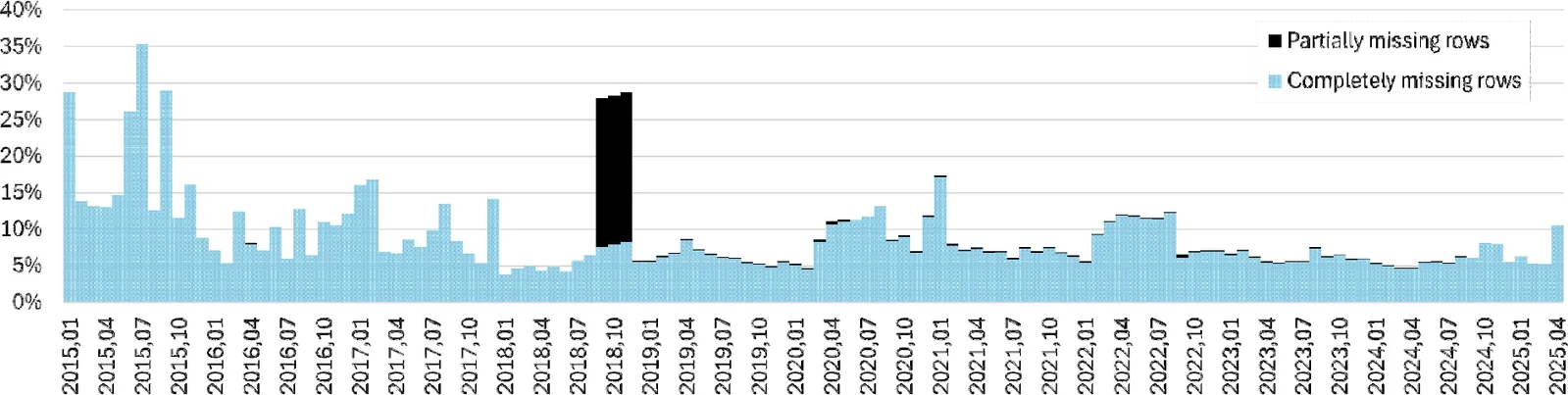
Light-blue bars are the percentage of completely missing rows within a year-month partition. Stacked on those, black bars are the percentage of partially missing rows within the same partitions.

Although partially missing rows account for only 0.59% on average across year-month partitions, Fig. 3 reveals a marked anomaly in September, October and November 2018, when this ratio rises to approximately 20.4% in each of the three consecutive months. The current gap summary indicates that this event is broad and fragmented, affecting 3,928 sensors and comprising 1,725,353 partial-gap episodes, most of them short to medium in duration. Moreover, these three months account for the large majority of all partially missing rows in the dataset, which suggests that the overall predominance of the 010 missingness pattern shown in Table 4 is largely driven by this period; that is, rows where sensor_occupancy is missing while traffic_intensity and avg_speed remain available. The anomaly is also already present in the original source data and is therefore not an artifact introduced by the GO-MO curation process. Taken together, the abrupt onset in September 2018, its persistence through November, and its sharp reduction in December suggest that the anomaly is more consistent with a temporary data outage or preprocessing issue at the City Council level prior to publication than with ordinary independent sensor outages, although the precise upstream cause cannot be determined from the available data alone.

**Table 4:** The **is_interpolated (I)** field indicates which of the three traffic metrics (intensity, occupancy, or average speed) were interpolated during curation (1 = missing, 0 = present).

I	# rows	Percent (%)	# sensors
0 0 1	866,947	$5.7\times {10}^{-2}$	782
0 1 0	6,831,156	$44.8\times {10}^{-2}$	3786
0 1 1	900,237	$5.9\times {10}^{-2}$	3077
1 0 0	19,357	$0.1\times {10}^{-2}$	950
1 0 1	35	$0.2\times {10}^{-6}$	17
1 1 0	33,742	$0.2\times {10}^{-2}$	1054
1 1 1	130,406,318	$856.2\times {10}^{-2}$	5148

**# rows** refers to the number of rows with the missing pattern on the left, **Percent (%)** is Num.rows divided by the total number of rows in the dataset, and **# sensors** is the count of unique sensors that are associated with each missing data pattern.

**Fig. 4:**
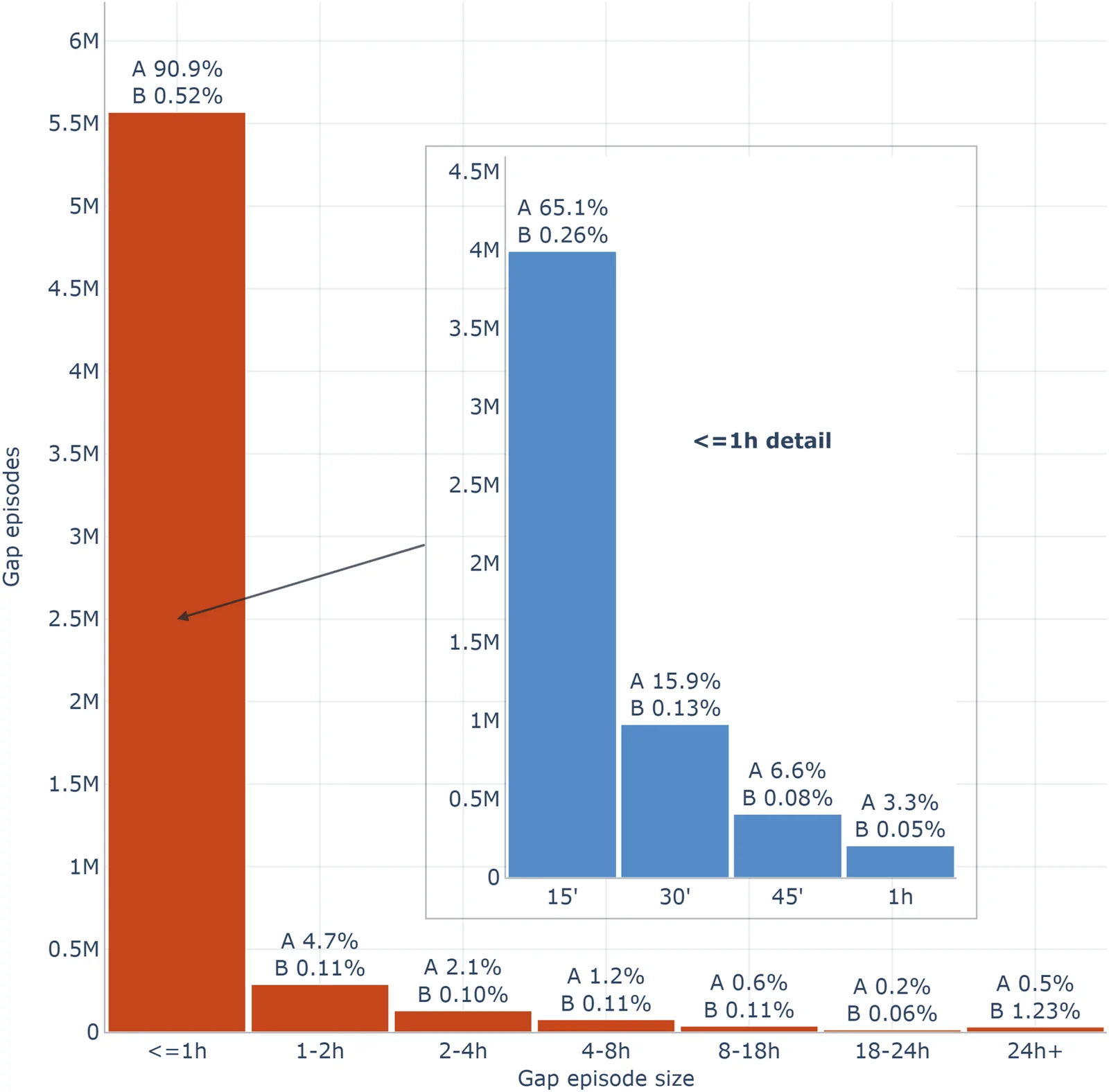
Distribution of fully missing gap episode durations. Labels marked A denote the percentage of fully missing gap episodes in each duration bin, while labels marked B denote the percentage of all dataset rows covered by fully missing intervals in that bin. The inset expands the ≤1 h bin into 15-, 30-, 45-, and 60-minute episodes.

To address missing values, we impute both fully and partially missing data using linear interpolation. This choice was motivated by three practical considerations. First, most missing-data episodes are short relative to the 15-minute sampling resolution of the dataset, as shown in Fig. 4: the median fully missing episode lasts 15 minutes, and 90.86% of fully missing episodes last one hour or less. Therefore, for the dominant class of short interruptions, linear interpolation provides a simple and reproducible approximation between neighbouring observed values. Second, the scale of the dataset, with more than 1.5 billion rows, makes more complex model-based or multivariate imputation methods computationally expensive as a general-purpose curation step. Third, such methods would introduce additional modelling assumptions that may not be appropriate for all downstream tasks.

Nevertheless, traffic is a highly dynamic and non-linear process, and linear interpolation may smooth peaks and troughs or distort temporal patterns, especially during long outages or periods with elevated missingness. For this reason, interpolated values should not be interpreted as direct sensor observations. To make this limitation explicit, a new field, is_interpolated, is added to the database to track modified records. This field contains a three-bit flag where each bit corresponds to traffic_intensity, sensor_occupancy, and avg_speed, respectively. A bit value of 1 indicates that the corresponding field has been interpolated, while 0 denotes original, unmodified data. This allows users to retain the complete version of the dataset, exclude interpolated values, use them as masks during model training, or apply alternative task-specific imputation methods. Table 4 provides a detailed summary of the missing-data patterns. Additionally, to quantify the uncertainty introduced by this imputation step, we further perform a masking-and-reconstruction analysis in the Technical Validation section.

In addition to the temporal analysis of missingness, we assess the spatial distribution of interpolated rows by computing sensor-level interpolation rates, aggregating interpolated rows on a 1 km grid, and evaluating spatial autocorrelation with Moran’s I.

**Fig. 5:**
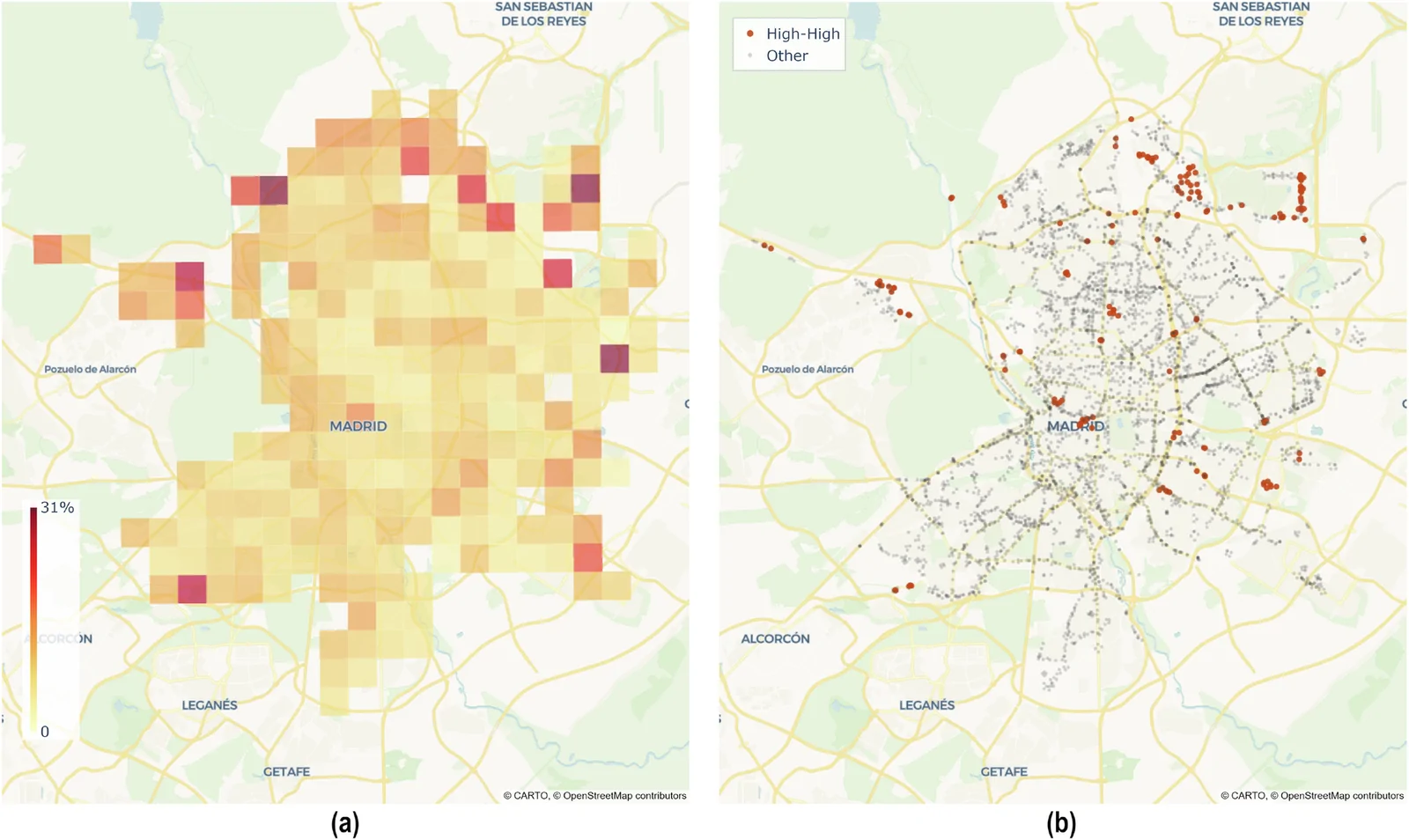
Spatial distribution of missing data. (**a**) Fully interpolated rows aggregated on a 1 km grid. (**b**) Local spatial clustering of sensor-level interpolation rates. High-High points denote sensors with high interpolation rates surrounded by neighbouring sensors that also exhibit high interpolation rates. Together, both panels show that the interpolation burden is spatially heterogeneous and presents localized hotspots rather than a uniform geographic pattern.

Figure 5(a) shows that missing data are not uniformly distributed across the city. Instead, missingness exhibits a heterogeneous spatial pattern, with several localized hotspots and broader areas with comparatively low interpolation rates. The grid analysis covers 232 occupied cells and indicates a clear but not extreme concentration of missingness: the top 10 cells account for 12.8% of all fully interpolated rows, and the top 50 cells account for 47.6%. This suggests that the burden of interpolation is spatially concentrated, but not restricted to only a handful of locations.

This pattern is further supported by the local spatial autocorrelation analysis shown in Fig. 5(b). Using sensor-level rates of fully interpolated rows over 5,085 sensors, the global Moran’s I is 0.1402, compared with an expected value close to −0.0002 under spatial randomness. This result is statistically significant under permutation testing (p=0.002, 499 permutations), indicating positive spatial autocorrelation in the distribution of fully interpolated data. The local Moran analysis identifies 199 High-High clusters, 24 Low-Low clusters, 58 High-Low spatial outliers, and 75 Low-High outliers. Hence, areas with comparatively higher interpolation burden are spatially localized and identifiable, providing a geographic characterization of missing-data quality across the released dataset.

### Graph generation

Traffic is inherently a spatio-temporal phenomenon that is often modeled as a graph. This paper introduces two graph representations extracted from traffic data: one for routes between nodes and another for the road network.

#### Routes Graph (RG)

Previous datasets (e.g.^14,16^) commonly build graphs based on pairwise distances between sensors. Following this methodology, we construct RG, where edge weights are derived from the shortest route distances between sensor pairs, computed using the Open Source Routing Machine (OSRM) (https://project-osrm.org/). Specifically, we calculate routes only between sensors that are separated by less than 500 meters. The resulting distances are normalized using Eq. (2),

2 $$w=exp\left(-\frac{{d}^{2}}{\sigma }\right),$$

where *w* represents the weight assigned to the edge, *d* denotes the route distance between two sensors, and *σ* is the standard deviation of all computed route distances. In summary, RG is a directed graph comprising 5,157 nodes and 135,428 edges, with an average node degree of 52.52. Every node represents a traffic sensor and therefore has an attribute containing the sensor ID; and every edge contains an attribute with the computed weight *w*.

#### Road network graph (RNG)

Additionally, we construct the RNG, which reflects the structure of Madrid’s road network by means of OpenStreetMap. The area of interest is defined by the bounding box formed by the northern-most, southern-most, eastern-most, and western-most sensors with available data. In RNG, each edge represents a road segment and each node corresponds to an intersection where two or more roads converge. The graph is constructed using the OSMnx Python package^23^, and is implemented as a MultiDiGraph; such that every one-way street from node A to node B is a directed edge from A to B, while a two-way street is represented by two directed edges: one from A to B and another from B to A. Moreover, several edges from A to B are possible, e.g. in inner-city highways with a service road. As a result, RNG has 35,304 nodes and 69,362 edges with an average node degree of 3.92, of which only 5,157 (7.21%) contain traffic sensors.

We note that these two graph representations are intended to support different kinds of analyses. RG follows the type of sensor-to-sensor graph commonly adopted in the traffic forecasting literature, where relations between sensors are modeled through route-based connectivity. By contrast, RNG preserves the physical road-network layout, with intersections as nodes and road segments as edges. We therefore release RNG as a complementary representation for problems that require explicit access to the underlying road topology and segment-level metadata, such as data-driven high-fidelity simulations of traffic conditions, digital twins of the road network, or analyses involving unsensed parts of the infrastructure. These tasks are not directly supported by a classic RG and would otherwise require reconstructing the missing road-network structure from external geospatial sources.

### Ethics and privacy

This study is based exclusively on publicly available, aggregated traffic measurements published by the Open Data Portal of the Madrid City Council. Formal ethics approval was not required because the released GO-MO dataset does not contain human-subject data or personally identifiable information. Although part of the source traffic-sensing infrastructure includes cameras equipped with computer vision systems, GO-MO only releases aggregated sensor-level traffic measurements at 15-minute intervals, together with sensor metadata and graph representations. The released dataset does not include images, videos, licence plates, vehicle-level identifiers, individual trajectories, or any other information that could be used to identify individual persons or vehicles.

## Data Record

The GO-MO dataset is published as a Hugging Face Dataset^24^ under a CC BY 4.0 license. It comprises four elements: 1) a collection of **ten yearly CSV files,** 'traffic_data_YYYY.csv', where YYYY ranges from 2015 to 2024 (one CSV file per year). 2) the traffic_sensors.csv file 3) the Route Graph (RG) and 4) the Road Network Graph (RNG).

All of them are linked by the sensor ID, which is a field in the CSV files, an attribute of every node in RG and an attribute of those specific nodes containing a sensor in RNG.

The CSV files are obtained by means of querying the relational database created for the extraction and transformation processes. Table 5 summarizes all the fields resulting from this query. The fields in traffic_data_YYYY.csv and in traffic_sensors.csv are the *essentially* the same as those described for traffic_data (from Table 2) and traffic_sensors (from Table 3) respectively.

**Table 5:** Fields in every CSV data file published in GO-MO.

traffic_data_YYYY.csv		traffic_sensors.csv	
sensor_id	as in Table 2	id	as in Table 3
entry_date	as in Table 2	element_type	{URB, Non-URB}
traffic_intensity	as in Table 2	district	as in Table 3
sensor_occupancy	as in Table 2	internal_code	as in Table 3
avg_speed	as in Table 2	name	as in Table 3
read_error	as in Table 2	utm_x	as in Table 3
→ is_interpolated	3-bit flag	utm_y	as in Table 3
		longitude	as in Table 3
		latitude	as in Table 3
		➜ lanes	Num. of lanes in that segment
		➜ max_speed	Speed limit in that segment
		➜ osm_name	OpenStreetMap street name

means “updated”, i.e., if the value was missing, it has been interpolated. ➜ indicates that the field is new with respect to the open source CSV files. YYYY refers to the year, ranging from 2015 to 2024.

However, the three fields marked with × in Table 2 have been removed in the published files. Specifically, element_type is omitted because it is duplicated in the sensors metadata CSV. The road_load field is excluded because its purpose is to support decision-making regarding the duration of traffic signal phases. Its calculation depends on parameters that are specific for each traffic light and time period and have not been published by the city council. Finally, integration_period is redundant since all the data is aggregated at 15-minute intervals. On the other hand, the fields lanes, max_speed and osm_name, together with is_interpolated are also included in the final published files.

Regarding the graphs, both RG and RNG are released as Python NetworkX (https://networkx.org/) multi-directed graphs in pickle format, named as routes-graph.pkl and road-network-graph.pkl respectively. We include the following metadata as edge attributes and provide brief description of each one in Tables 6, 7.

1. OSMid, is the OpenStreetMap key of that segment;
2. length, geometry and reversed are produced by OSMnx from OpenStreetMap information for every given edge;
3. access, bridge, est_width, highway, junction, lanes, maxspeed, name, oneway, ref, tunnel, width, lanes:backward, lanes:forward, lanes:psv:backward lanes:psv:forward, maxspeed:lanes:backward and maxspeed:lanes:forward are attributes of an edge directly provided by OpenStreetMap (also known as ‘tags’).
4. To associate the CSV files with the RNG, we perform reverse geocoding using each sensor’s UTM coordinates (utm_x and utm_y) to identify the nearest road segment. The closest edge to each sensor is annotated with another attribute containing the sensor’s unique identifier sensor_id.

**Table 6:** OSMnx attributes.

Attribute name	Description
OSMID	The open street maps id of the road. i.e. OSMID = 1287001472. Can be used to locate the OSM road segment as: https://www.openstreetmap.org/way/1287001472
length	The road segment length in meters, according to the road geometry
geometry	The road segment physical geometry as a Shapely Multipolygon
reversed	Indicates whether the edge represents reversed direction from the original OSM way. Consider a bidirectional street with nodes u and v.
	OSMnx creates two edges:
	• (u, v) with reversed = False
	• (v, u) with reversed = True
	So for the reversed = True edge, the nodes are reversed from the original OSM way.

**Table 7:** OSM attributes.

Attribute name	Description	Number of edges	Doc. Link
access	The access key describes restrictions on the use of highways and other transportation routes (railways, waterways), as well as facilities such as buildings, entrances and barriers, shops, amenities and leisure grounds.	880	https://wiki.openstreetmap.org/wiki/Key:access
bridge	The bridge key is a property to describe that a way is on a bridge. It can be combined with several types of ways such as roads, railways, path canals, pipelines or similar. The value of the key is used to indicate the type of bridge.	830	https://wiki.openstreetmap.org/wiki/Key:bridge
est_width	The estimated width of a feature. Values and documentation are as “width” attribute.	12	https://wiki.openstreetmap.org/wiki/Key:est_width
highway	The key highway is the main key used for identifying any kind of road, street or path. The value of the key helps indicate the type and importance of the highway within the road network.	69362	https://wiki.openstreetmap.org/wiki/Key:highway
junction	This key describes how a specific junction is constituted. Two possible values, “roundabout” for circular (self-intersecting) road junctions where the traffic on the roundabout has always right of way. “circular” for roundabouts where traffic on the junction does not have complete right of way.	4838	https://wiki.openstreetmap.org/wiki/Key:junction
lanes	The lanes key value represents how many traffic lanes there are on a road. To tag individual lanes of a road, see the article about the lanes suffix. Note that it is valid to tag lanes count also in case where lanes are not marked with paint on the surface.	37845	https://wiki.openstreetmap.org/wiki/Key:lanes
lanes:backward	If the lanes on a two-way road are not distributed evenly between the driving directions, the keys lanes:forward = * and lanes:backward = * can be used in addition to the lanes tag. The key lanes:forward = * hereby refers to lanes which direction is equal to the direction of the OSM way, and lanes:backward = * to the opposite direction.	4397	https://wiki.openstreetmap.org/wiki/Key:lanes#Lanes_in_different_directions
lanes:forward	If the lanes on a two-way road are not distributed evenly between the driving directions, the keys lanes:forward = * and lanes:backward = * can be used in addition to the lanes tag. The key lanes:forward = * hereby refers to lanes which direction is equal to the direction of the OSM way, and lanes:backward = * to the opposite direction.	4458	https://wiki.openstreetmap.org/wiki/Key:lanes#Lanes_in_different_directions
lanes:psv:backward	The lanes:psv key can be used to mark the number of lanes reserved for public service vehicles on a way. The most common value is 1, when a psv lane exists. For a two-way road with dedicated psv lanes in both directions, the value would be 2.	422	https://wiki.openstreetmap.org/wiki/Key:lanes:psv
lanes:psv:forward	The lanes:psv key can be used to mark the number of lanes reserved for public service vehicles on a way. The most common value is 1, when a psv lane exists. For a two-way road with dedicated psv lanes in both directions, the value would be 2.	401	https://wiki.openstreetmap.org/wiki/Key:lanes:psv
maxspeed	The maxspeed tag is used on ways to define the maximum legal speed limit for general traffic on a particular road, railway, or waterway. By default, values will be interpreted as kilometers per hour. For roads with multiple lanes, with different speed limits for different lanes, the different speed values are separated by ‘|‘ character, like in ‘50|50|30’.	25875	https://wiki.openstreetmap.org/wiki/Key:maxspeed
maxspeed:lanes:backward	If the speed limits should be specified for each driving direction separately the key maxspeed:lanes:forward resp. maxspeed:lanes:backward can be used, whereas lanes:forward applies to the speed limit of the lanes for the same direction as the OSM way and lanes:backward to the speed limit of the lanes of the opposite direction. Same rules as lanes key are used.	200	https://wiki.openstreetmap.org/wiki/Key:maxspeed#Lanes
maxspeed:lanes:forward	If the speed limits should be specified for each driving direction separately the key maxspeed:lanes:forward resp. maxspeed:lanes:backward can be used, whereas lanes:forward applies to the speed limit of the lanes for the same direction as the OSM way and lanes:backward to the speed limit of the lanes of the opposite direction. Same rules as lanes key are used.	198	https://wiki.openstreetmap.org/wiki/Key:maxspeed#Lanes
name	This key is set to the primary name of the feature in the real world. As a rule of thumb, the primary name is the most obvious name of the feature, the one that end users expect data consumers to expose in a label or other interface element. The usual sources of primary names are: • The most prominent name on a sign posted on the feature itself, especially for a feature in the built environment bullet The name of the feature on a sign elsewhere, such as a guidepost bullet Common or official usage, especially for a feature in the natural environment	62288	https://wiki.openstreetmap.org/wiki/Key:name
oneway	The one-way key is used to indicate the access restriction on highways and other linear features for vehicles as appropriate. This means that this key should be used when this way can only be used in one direction by vehicles.	69362	https://wiki.openstreetmap.org/wiki/Key:oneway
ref	“ref” stands for “reference” and is used for reference numbers or codes. Common for roads, highway exits, routes, entrances to big buildings etc. It is also used for shops and amenities that are numbered officially as part of a retail brand or network respectively.	2017	https://wiki.openstreetmap.org/wiki/Key:ref
tunnel	The tunnel key is used for roads, railway lines, canals, etc. that run in a tunnel. It contains “yes” to indicate the road runs in a tunnel. A value “building_passage” indicates a tunnel that runs through/under a building.	629	https://wiki.openstreetmap.org/wiki/Key:tunnel
width	The key width describes the actual width of a way or other feature. Values will be interpreted as meters.	476	https://wiki.openstreetmap.org/wiki/Key:width

**Number of edges** is the count of how many edges included in RNG contain the attribute described in a given row. **Doc. Link** links to the official OSM documentation.

## Data Overview

The traffic sensor network is deployed across the city of Madrid, with sensors installed on the distribution highways surrounding the city center (non-urban sensors), and a large number of inner-city streets and major access roads (urban sensors). The dataset reveals seasonal patterns and temporal trends driven by traffic in cities with more than three million inhabitants. These cities are typically major economic and employment hubs, which attract a large floating population. Specifically, Madrid has a population of approximately 3.45 million inhabitants and a floating population of around 990,000 people who commute daily from surrounding municipalities using both public and private transport. In total, 2,150,000 people work or study in the city. During a typical working month (using October 2023 as a reference) approximately 13,000,000 trips are recorded, of which 6,000,000 are made by private vehicles^25^.

**Fig. 6:**
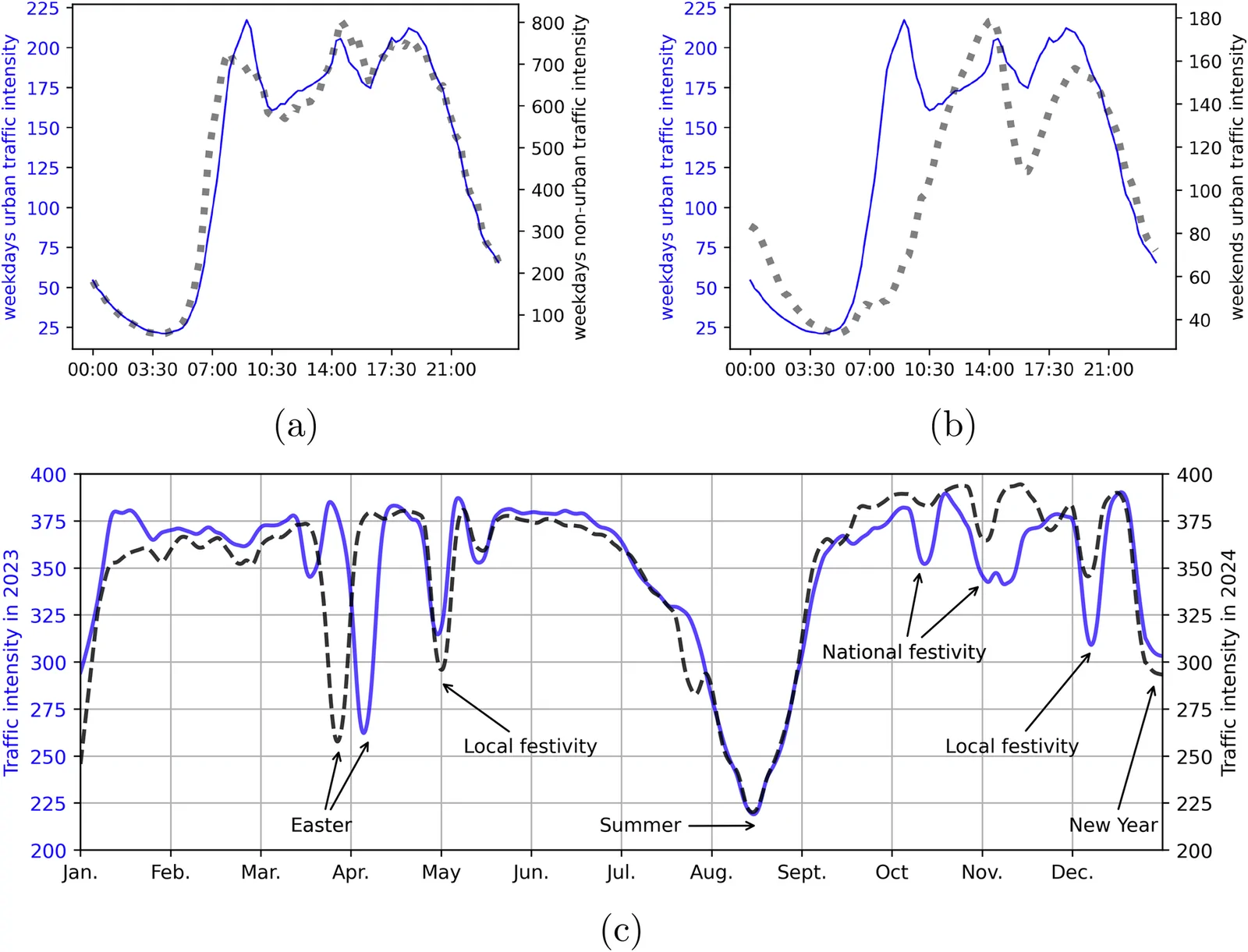
(**a**) Urban traffic intensity in weekdays -left Y, solid line- vs. non urban -right Y, dashed line- (**b**) Urban traffic intensity in weekdays -left Y, solid line- vs. weekends -right Y, dashed line- (**c**) Daily average traffic intensity in 2023 -left Y, solid line- vs. 2024 -right Y, dashed line.

Figure 6(a) shows the average traffic intensity patterns in weekdays (Monday to Friday), captured at 15-minute intervals, in 2023 by urban (left Y-axis) and non-urban (right Y-axis) sensors. The pattern reveals typical commuting behaviors, with peaks in the morning, lunchtime (which in Madrid is usually between 1:30 pm and 3:00 pm) and evening. This pattern changes substantially during weekends (Saturday and Sunday), especially in the morning, when the weekday peak disappears, as shown in Fig. 6(b), which compares the average traffic intensity patterns in 2023 for urban traffic on weekdays (left Y-axis) and weekends (right Y-axis). The other two peaks appear later in the day and are associated to leisure activities such as lunch and dinner outings.

On the other hand, a view to a full year of traffic reveals seasonal patterns similar to other western cultures such as Christmas and New Year, Easter and Summer holidays, together with other national and local festivities that significantly reduce the traffic intensity. These patterns are shown in Fig. 6(c), which presents the average traffic intensity in 2023, captured at 15-minute intervals (left Y-axis) against the same during 2024 (right Y-axis). Deviations can be observed in Easter, which occur on different calendar weeks each year; while national and local festivities may differ more when they are before or after the weekend.

## Technical Validation

### Interpolation-error analysis

To quantify the uncertainty introduced by the imputation step, we performed a masking-and-reconstruction experiment on originally observed traffic_intensity values from the full 2015–2024 period. For each gap-duration class, up to 100 gaps per sensor were sampled from all eligible sensors, temporarily removed, reconstructed using the same linear interpolation procedure applied in GO-MO, and compared with their original observed values. Errors are computed at the imputed-record level. Because traffic intensity varies substantially across sensors, time of day, weekdays and weekends, and seasons, absolute errors in vehicles/hour do not have the same relative meaning under all traffic regimes. We therefore also report the weighted absolute percentage error (WAPE), computed as the sum of absolute reconstruction errors divided by the sum of the corresponding observed traffic intensities within each gap-duration class. This provides a scale-normalized measure of interpolation error.

Table 8 shows that the interpolation error increases with gap duration. Short gaps are reconstructed with moderate error: single 15-minute gaps have a MAE of 43.31 vehicles/hour, a median absolute error of 21.50 vehicles/hour, and a WAPE of 10.96%. Errors remain similar for gaps up to one hour, with WAPE increasing only moderately to 13.09%. For longer gaps, the error increases more substantially, reaching a MAE of 142.45 vehicles/hour and a WAPE of 37.16% for 6–12 hour gaps, and 211.21 vehicles/hour and 56.91% for 12–24 hour gaps. Gaps longer than 24 hours show the largest errors, with a MAE of 253.38 vehicles/hour, a WAPE of 66.74%, and a 95th percentile absolute error of 986.43 vehicles/hour. This pattern is expected because linear interpolation assumes a smooth transition between the observations bounding the missing interval, an assumption that becomes less reliable as the duration of the gap increases.

**Table 8:** Interpolation error for traffic_intensity, estimated by masking originally observed records from the full 2015–2024 period and reconstructing them with the same linear interpolation procedure used in GO-MO.

Gap duration	# sensors	# gaps	# points	MAE	RMSE	WAPE (%)	Median AE	95th pct. AE
15 min	5,064	505,844	505,844	43.31	113.06	10.96	21.50	150.00
30 min	5,064	505,802	1,011,604	46.56	109.56	11.81	22.67	162.33
45 min	5,062	505,773	1,517,319	49.60	122.66	12.55	24.00	174.00
1 h	5,061	505,730	2,022,920	51.75	125.43	13.09	24.60	182.00
1–3 h	5,061	505,981	4,113,895	67.27	178.85	16.98	29.33	244.00
3–6 h	5,060	505,574	8,922,376	98.11	251.73	24.84	41.57	365.81
6–12 h	5,054	505,137	17,004,664	142.45	316.86	37.16	61.16	538.93
12–24 h	5,048	504,690	31,914,693	211.21	439.18	56.91	87.84	811.33
>24 h	4,996	499,060	86,246,940	253.38	501.25	66.74	107.86	986.43

Gap-duration classes match those used in the missing-gap duration analysis. Up to 100 gaps per sensor and duration class were sampled. Errors are reported in vehicles/hour, except for WAPE, which is reported as a percentage of the observed traffic intensity in each gap-duration class.

### Forecasting benchmark

In this paper we leverage LargeST^16^, a large-scale traffic forecasting benchmark that provides both a dataset and code for benchmarking spatio-temporal graph models. While its dataset primarily consists of California highway traffic, which differs significantly from the urban traffic patterns in GO-MO, in this work we only use its implemented framework to carry out a comparison of the models included. Specifically, we evaluate 11 models grouped into six families:

**Recurrent neural networks:** LSTM^26^.

**GNNs with recurrent components:** DCRNN^14^ and AGCRN^27^.

**Spatio-temporal convolutional models:** STGCN^28^ and GWNET^28^.

**Attention-based architectures:** ASTGCN^29^ and STTN^30^.

**Neural ODE-based models:** STGODE^31^.

**Dynamic graph-based models:** DSTAGNN^32^, DGCRN^33^, and D2STGNN^34^.

With them, we provide a comprehensive baseline for traffic intensity forecasting at horizons of 3, 6, and 12 time steps ahead. Since GO-MO is sampled at 15-minute intervals, these correspond to 45, 90, and 180 minutes ahead, respectively. For the experiments, we select the latest year (2024), reshaped such that each timestamp corresponds to a single row, and the traffic intensity values from all sensors active in 2024, resulting in 4,769 columns.

Besides, we extract the subset that only keeps those sensors with element_type = Non-URB, which reduces the number of columns to 287. Thus, for the sake of experiments, we have two working datasets, *Full Dataset* (FDS) and *Non-Urban Dataset* (NUDS), both with 35,136 rows, which are split into training, validation, and testing sets using a 60/20/20 ratio, i.e.: 21,067 rows for training, 7,022 for validation, and 7,024 for testing. Additionally, since LargeST requires a route graph, all the experiments are carried out using RG.

Experiments were conducted on two distinct hardware configurations, each dedicated to a specific subset of tasks depending on the GPU memory requirements. The experiments with FDS were executed on a high-performance server equipped with 384 AMD EPYC 9654 96-core CPUs, 1.5 TB of RAM, and 4 NVIDIA H100 GPUs, each with 95 GB of memory; whereas experiments with NUDS were run on a separate server featuring 256 AMD EPYC 7742 64-core CPUs, 1 TB of RAM, and 8 NVIDIA A100 GPUs with 80 GB of memory each.

Table 9 presents the baseline results for forecasting experiments conducted on both subsets of the dataset. Among the evaluated models, DGCRN^33^ and D^2^STGNN^34^ consistently outperform the others across nearly all evaluation metrics in the non-urban sensors dataset. In the full dataset group, GWNET^28^ outperforms all models across all metrics. DGCRN^33^ and D^2^STGNN^34^ are too large for the FDS so no results are provided for them.

**Table 9:** Baseline experiments results.

Subset	Model	Horizon 3			Horizon 6			Horizon 12			Average		
		MAE	RMSE	MAPE	MAE	RMSE	MAPE	MAE	RMSE	MAPE	MAE	RMSE	MAPE
NUDS	LSTM	38.634	68.728	22.07%	51.369	91.560	27.59%	67.808	121.235	35.76%	50.945	90.736	27.73%
	DCRNN	33.959	61.587	18.49%	39.891	72.343	21.47%	47.300	87.362	25.50%	39.277	71.382	21.31%
	AGCRN	31.403	58.201	19.05%	36.182	68.187	21.93%	40.851	78.313	**22.82%**	35.402	66.560	20.96%
	STGCN	34.254	61.548	19.90%	40.244	72.356	22.43%	46.830	82.892	25.48%	39.702	70.823	22.29%
	GWNET	30.752	**56.547**	20.07%	36.091	66.688	23.31%	41.831	77.508	23.80%	35.367	65.184	21.96%
	ASTGCN	40.849	72.057	21.23%	48.036	84.855	25.34%	56.973	97.251	31.06%	46.271	80.935	24.72%
	STTN	32.840	59.872	17.84%	38.066	70.689	**20.51%**	44.099	81.735	25.30%	37.329	68.799	20.50%
	STGODE	33.189	60.155	18.32%	38.530	70.764	21.33%	43.573	80.831	24.80%	37.529	68.778	20.86%
	DSTAGNN	36.584	64.415	22.50%	43.090	75.828	23.48%	52.190	90.075	32.31%	42.393	74.153	24.47%
	DGCRN	**30.636**	56.664	18.31%	**35.144**	**65.708**	20.61%	**40.268**	**76.131**	23.74%	**34.510**	**64.335**	**20.38%**
	D2STGNN	32.208	59.198	**17.46%**	38.143	69.790	20.75%	45.238	83.865	25.47%	37.514	68.451	20.69%
FDS	LSTM	11.288	25.554	28.27%	14.209	31.962	33.43%	18.288	40.905	43.38%	14.225	31.993	34.09%
	DCRNN	10.554	23.994	28.34%	12.502	28.160	32.63%	15.394	34.600	39.99%	12.518	28.240	33.01%
	AGCRN	10.200	25.029	29.57%	11.508	28.363	32.12%	12.975	31.976	35.54%	11.419	28.078	31.85%
	STGCN	12.588	30.437	33.42%	14.131	33.402	35.52%	15.913	36.621	38.73%	14.047	33.177	35.46%
	GWNET	**9.944**	**22.859**	**26.49%**	11.384	**25.960**	30.98%	13.187	**29.667**	**34.25%**	11.295	**25.704**	30.64%
	ASTGCN	12.875	28.308	34.31%	16.718	36.616	42.67%	21.935	48.707	55.91%	16.688	36.769	43.18%
	STTN	10.107	23.463	26.51%	11.401	27.142	**28.35%**	13.100	31.429	36.28%	11.317	26.745	**29.80%**
	STGODE	10.030	23.266	27.38%	**11.118**	26.855	29.93%	**12.590**	31.048	34.73%	**11.074**	26.611	30.13%
	DSTAGNN	11.732	26.514	30.97%	14.195	31.997	36.22%	17.487	39.108	66.21%	14.134	31.853	38.53%

Best performance is bold. NUDS include only non-urban sensors, whereas FDS include all sensors.

Table 10 presents a comparison of the baseline models in terms of efficiency. More recent architectures such as ASTGCN, STGODE, and DSTAGNN tend to have a significantly larger number of parameters, which results in increased training time and memory consumption. However, this added complexity often translates into improved performance. Notably, the inference time differences between models are less pronounced, suggesting that despite their heavier architectures, newer models remain competitive in deployment scenarios.

**Table 10:** Efficiency comparison for the models in the baseline.

Subset	Model	Num. param	Batch size	Epochs	Duration (h)	Train time (s)	Inference time (ms)
NUDS	LSTM	97,932	64	100	0.41	14.66	0.39
	DCRNN	373,313	64	100	4.75	170.70	3.10
	AGCRN	756,290	64	100	1.34	48.05	0.71
	STGCN	287,884	64	100	0.63	22.66	0.62
	GWNET	302,584	64	100	0.81	29.03	0.53
	ASTGCN	402,285	64	100	1.10	39.54	0.71
	STTN	86,284	64	100	1.22	43.65	0.81
	STGODE	713,784	64	100	1.65	51.69	0.95
	DSTAGNN	1,354,476	64	100	1.47	52.89	0.88
	DGCRN	208,529	64	100	4.10	147.52	2.80
	D2STGNN	396,004	64	50	1.84	65.98	2.45
FDS	LSTM	97,932	64	100	2.77	99.37	2.14
	DCRNN	373,313	64	100	49.37	1774.39	37.30
	AGCRN	797,150	64	100	24.33	874.65	15.26
	STGCN	2,379,916	64	100	4.49	161.05	5.31
	GWNET	384,304	64	100	19.78	710.73	12.89
	ASTGCN	76,847,259	16	100	45.43	1633.51	22.80
	STTN	347,788	8	100	49.90	1777.33	27.62
	STGODE	860,880	8	100	56.46	799.22	15.48
	DSTAGNN	84,954,036	8	47	38.24	1372.81	47.49

**Num. param**: total number of parameters of the model, **Batch size**: size of the batch used for training, **Epochs:** number of epochs of training, **Duration**: total number of hours of training, **Train time**: train time in seconds for each epoch, **Inference time**: time of inference for a single sample in milliseconds.

## Data Availability

The GO-MO dataset is published as a Hugging Face Dataset under a CC BY 4.0 license. It comprises four elements: 1) a collection of ten CSV files, ‘traffic_data_YYYY.csv’ where YYYY ranges from 2015 to 2024 (one CSV file per year). 2) the traffic_sensors.csv file 3) the Route Graph (RG) and 4) the Road Network Graph (RNG). It can be accessed at (https://doi.org/10.57967/hf/7201).

## Code availability

The source code used to generate the auxiliary graph formats, derive road-network line graphs, export adjacency matrices, convert graphs to PyTorch Geometric format, and generate model-ready data files for the validation experiments is available in the public GitHub repository https://github.com/dmariaa/gomo-traffic. The fixed version associated with this manuscript is release v1.0.0, available at https://github.com/dmariaa/gomo-traffic/releases/tag/v1.0.0, corresponding to commit 7eada8e. The code is distributed under the MIT license.

The repository includes installation and execution instructions. In brief, users can install the required dependencies with pip install -r requirements.txt, add the src/ directory to PYTHONPATH, and run the documented scripts from the repository root. The code was developed for Python 3.10+, and the required Python packages are listed in the repository requirements.txt file. The README documents the scripts used to generate LargeST-compatible data files, export graph representations, construct line graphs, compute graph statistics, and convert NetworkX graphs to PyTorch Geometric data objects.
